# Genome-wide comparative transcriptome analysis of CMS-D2 and its maintainer and restorer lines in upland cotton

**DOI:** 10.1186/s12864-017-3841-0

**Published:** 2017-06-08

**Authors:** Jianyong Wu, Meng Zhang, Bingbing Zhang, Xuexian Zhang, Liping Guo, Tingxiang Qi, Hailin Wang, Jinfa Zhang, Chaozhu Xing

**Affiliations:** 1 State Key Laboratory of Cotton Biology, Institute of Cotton Research, Chinese Academy of Agricultural Sciences, Key Laboratory for Cotton Genetic Improvement, Ministry of Agriculture, 38 Huanghe Dadao, Anyang, 455000 China; 2 Department of Plant and Environmental Sciences, New Mexico State University, Las Cruces, NM 88003 USA

**Keywords:** Upland cotton, CMS-D2, RNA-seq, Restorer gene, Circadian rhythm

## Abstract

**Background:**

Cytoplasmic male sterility (CMS) conferred by the cytoplasm from *Gossypium harknessii* (D2) is an important system for hybrid seed production in Upland cotton (*G. hirsutum)*. The male sterility of CMS-D2 (i.e., A line) can be restored to fertility by a restorer (i.e., R line) carrying the restorer gene *Rf1* transferred from the D2 nuclear genome. However, the molecular mechanisms of CMS-D2 and its restoration are poorly understood.

**Results:**

In this study, a genome-wide comparative transcriptome analysis was performed to identify differentially expressed genes (DEGs) in flower buds among the isogenic fertile R line and sterile A line derived from a backcross population (BC_8_F_1_) and the recurrent parent, i.e., the maintainer (B line). A total of 1464 DEGs were identified among the three isogenic lines, and the *Rf1*-carrying Chr_D05 and its homeologous Chr_A05 had more DEGs than other chromosomes. The results of GO and KEGG enrichment analysis showed differences in circadian rhythm between the fertile and sterile lines. Eleven DEGs were selected for validation using qRT-PCR, confirming the accuracy of the RNA-seq results.

**Conclusions:**

Through genome-wide comparative transcriptome analysis, the differential expression profiles of CMS-D2 and its maintainer and restorer lines in Upland cotton were identified. Our results provide an important foundation for further studies into the molecular mechanisms of the interactions between the restorer gene *Rf1* and the CMS-D2 cytoplasm.

**Electronic supplementary material:**

The online version of this article (doi:10.1186/s12864-017-3841-0) contains supplementary material, which is available to authorized users.

## Background

Cotton is the most important fiber crop and an important oil-producing crop worldwide. As in other crop plants, utilization of heterosis is an important way to improve yield in cotton production. To date, most commercial cotton hybrids have been produced by artificial emasculation and pollination (AEP) in China [1] and India (http://www.cicr.org.in/), which is a time-consuming, labor-intensive and costly process. In addition, the purity of hybrid seeds produced by AEP cannot be guaranteed as some artificial emasculation may not completely remove the pollen. The cytoplasmic male sterility (CMS) system is an ideal tool for hybrid seed production, and it has been widely used to facilitate the use of heterosis in many crops [2]. CMS-D2 is one of the two major types of CMS [3–6] in cotton and has contributed to cotton heterosis utilization. *Rf1* is the restorer gene and can recover the fertility of CMS-D2. Considering the importance of the CMS and restoration system, numerous molecular mapping studies have been conducted on of *Rf1* in cotton [7–13]. Recently, a backcross population (BC_8_F_1_) with plants distinguished as male fertile (F) or sterile (S) was generated and used to map the *Rf1* gene by our group [14]. However, there have been few studies on the molecular mechanism of the restorer gene.

Over the past several years, next-generation sequencing (NGS) has been used in numerous research areas, resulting in high-throughput production of massive DNA and RNA data [15]. As a powerful tool for studying global transcriptional networks, transcriptome sequencing provides high-resolution data and has been widely used in many crops. In cotton, it has been used to study boll development [16], fiber development [17–19], leaf senescence [20], gland morphogenesis [21], abiotic stress responses [22–24], biotic stress responses [25, 26], RNA editing in relation to CMS-D8 [27], and genic male sterility [28]. Differential display and gene chips were used to study the expression levels of differentially expressed genes (DEGs) associated with the fertility of CMS-D8 in cotton [29, 30]. However, the global gene expression patterns of CMS-D2 and its interaction with its restorer gene *Rf1* are still unknown. Now that the genome sequences of *G. raimondii* [31, 32], *G arboreum* [33], and *G hirsutum* [34, 35] have been published, gene annotation can be better performed, which will improve genome-wide transcriptome sequencing and analysis in cotton.

To better understanding the gene expression profiles affected by the restorer gene *Rf1* in Upland cotton with the CMS-D2 cytoplasm, RNA-seq by the Illumina NGS technology was used in this study to identify DEGs in flower buds of fertile (i.e., restorer R line) and sterile (i.e., CMS A line) plants of a backcross population (BC_8_F_1_) and its recurrent parent, i.e., the maintainer B line. GO and KEGG enrichment analysis showed that genes related to circadian rhythms were significantly affected by the presence of the restorer gene. The results from this study will serve as a foundation for further studies of the molecular mechanisms of interaction between the restorer gene *Rf1* and the CMS-D2 cytoplasm.

## Methods

### Plant materials

In our previous study [14], the sterile line ZBA with the CMS-D2 cytoplasm was crossed with the restorer line Zhonghui46, and then the maintainer B line (designated dB3) with the normal fertile Upland cotton (AD1) cytoplasm was used as the recurrent male parent to backcross with the F_1_ plants to construct a BC_8_F_1_ population. In this segregating population, the sterile plants (designated dZB3) were considered to be the CMS-D2 A line, and the fertile plants (designated dZK3) were considered to be the restorer R line. All materials were provided by Institute of Cotton Research (ICR), Chinese Academy of Agricultural Science (CAAS). The BC_8_F_1_ population and recurrent parent were grown in the Experimental Farm, ICR-CAAS, Anyang, Henan province, China. A randomized complete block design with three biological replications was used, and crop management practices followed local recommendations. On sunny days of about 30 °C, flowering buds of about 3 mm in length (at roughly the stage of male meiosis) were collected and combined from 50 plants for each genotype in each replication. All harvested samples were snap-frozen in liquid nitrogen and stored at −80 °C before use.

### RNA extraction, RNA-seq library construction and sequencing

Total RNA was isolated using the Sigma Spectrum Plant Total RNA kit (Sigma-Aldrich, USA) according to the manufacturer’s protocol. The concentration of each RNA sample was measured using a NanoDrop 2000 spectrophotometer (NanoDrop Technologies Inc., USA). Nine individual libraries (three samples for each of the three genotypes) were constructed with an Illumina RNA TruSeq kit (Illumina, USA) per the manufacturer’s instructions using 5 μg of total RNA. Subsequently, PCR amplification was performed using Phusion DNA polymerase (NEB, USA) for 15 PCR cycles, and f cDNA fragments of 300–500 bp were isolated from a 2% low range ultra agarose gel (Bio-Rad, USA). After quantification by TBS380 (Picogreen, Invitrogen, USA), the paired-end libraries were then sequenced using the Illumina HiSeq™ 2500 system (2 × 151 bp read length) at Shanghai Majorbio Bio-pharm Biotechnology Co., Ltd. (Shanghai, China).

### Data processing and expression analysis

SeqPrep (https://github.com/jstjohn/SeqPrep) and Sickle (https://github.com/najoshi/sickle) were used to remove low-quality reads (i.e., Q value <25), adapter sequences, reads with ambiguous bases (‘N’), and fragments of less than 20 bp in length. All clean reads were mapped to the *G. hirsutum* TM-1 reference genome (http://mascotton.njau.edu.cn/info/1054/1118.htm) using the TopHat software [36] which allowed no more than a 2-nucleotide mismatch. Gene annotation and expression quantification were performed using the software Cufflinks (http://cufflinks.cbcb.umd.edu/), and the FPKM (fragments per kilobase of exon per million fragments) method was used to identify DEGs based on a false discovery rate (FDR) of <0.05 and estimated absolute log_2_fold change > 1 between different genotypes. A heatmap was constructed using the web server ClustVis (http://biit.cs.ut.ee/clustvis/) with default parameters.

### Functional annotation

GO and KEGG functional annotations for the transcripts were retrieved using blast2go (http://www.blast2go.com/b2ghome) and blastx/blastp searches against the KEGG genes (http://www.genome.jp/kegg/genes.html) database, respectively. GO term and KEGG pathway enrichment analysis was performed on the significantly differentially expressed transcripts using the Goatools software (https://github.com/tanghaibao/Goatools) and KOBAS software (http://kobas.cbi.pku.edu.cn) [37], with a corrected *P*-value ≤0.05 as the threshold.

### Quantitative RT-PCR (qRT-PCR) validation

First-strand cDNA was generated from 1 μg total RNA from individual replications using a PrimerScript RT Reagent kit (Perfect Real Time, TaKaRa, Japan). Quantitative real-time RT-PCR was performed using SYBR® Premix Ex TaqTM (Perfect Real Time, TaKaRa, Japan) according to the manufacturer’s instructions. Primers for qPCR were designed using the Primer Express software (Applied Biosystems, Foster City, CA, USA), synthesized commercially (Tianyi Huiyuan Biotechnology, Beijing, China), and are shown in Additional file 1. PCR analysis was performed using a CFX96TM instrument (Bio-Rad, USA). Each reaction contained 2 μl cDNA template, 800 nM of each primer and 10 μl 2 × SYBR® Premix Ex TaqTM, with ddH_2_O to bring the final volume to 20 μl. The reaction was pre-denatured at 95 °C for 30 s, followed by 40 cycles of denaturation at 95 °C for 5 s, annealing at 58 °C for 20 s and extension at 72 °C for 30 s. A melting curve was generated for each sample at the end of each run to determine the specificity of the amplified products. Each gene was analyzed in triplicate, and controls without template were also included. Actin was used as an internal control. The threshold cycle (Ct) values of each reaction were determined automatically by the instrument software, and the relative amount of each gene to the internal control was calculated using the eq. 2^−ΔΔCt^, where ΔΔCt = (Ct target − Ct actin) sample X − (Ct target − Ct actin) sample 1. The whole assay protocol was repeated three times to ensure the reliability of the assay data. The standard deviations of the data were determined from the three independent experiments. The statistical significance of expression differences was analyzed using the Student’s *t*-test.

### Identification of SNPs

Single nucleotide polymorphism (SNP) loci for candidate genes were identified in the assembled transcript sequences using the Samtools (http://samtools.sourceforge.net/) and VarScan (http://varscan.sourceforge.net/) software.

## Results

### Transcriptome sequencing and mapping

In this study, near-isogenic A, B and R lines each comprising three individual biological samples of 3 mm-long flowering buds at the stage of male meiosis were used to construct cDNA libraries for a deep Illumina sequencing. After filtering the raw reads, 48,365,894, 46,208,878, and 40,915,284 clean reads for the three replicates of the maintainer B line (dB3), 35,886,986, 46,397,948, and 39,667,094 clean reads for the male sterile A line (dZB3) in the BC_8_F_1_ population, and 45,856,082, 42,6816,76, and 52,325,842 clean reads for the fertile restorer R line (dZK3) in the BC_8_F_1_ population were obtained (Additional file 2). More than 90% of these clean reads were mapped to the *G. hirsutum* TM-1 reference genome (Additional file 3). The deep RNA-seq had a 90.55–91.89% genome coverage of the predicted genes in Upland cotton. In total, 62,001 of the 70,478 predicted transcripts in the reference TM-1 genome were identified in this study and were used for a further analysis.

### GO and KEGG classification of the expressed genes

**Fig. 1:**
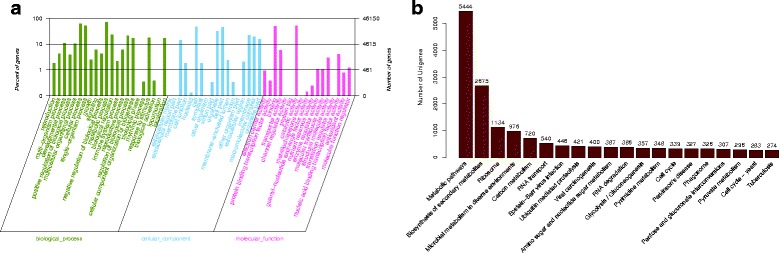
Gene ontology classification (**a**) and COG functional categories (**b**) of unigenes

Blast2go was used to retrieve the GO functional annotations, and the results showed that 46,150 of the 62,001 predicted transcripts were successfully assigned GO annotations within the three main GO categories and 57 sub-categories (Fig. 1a). ‘Metabolic process’ (32,285 genes; representing 69.9% of transcripts in the biological process category), ‘cellular process’ (28,157 genes; 61.0%), and ‘single-organism process’ (23,292 genes; 50.5%) had the highest numbers of genes in the biological process category. ‘Cell’ (21,221 genes; representing 46.0% of transcripts in the cellular component category), ‘cell part’ (20,897 genes; 45.3%) and ‘organelle’ (14,269 genes; 30.9%) had the most genes in the cellular component category. ‘Catalytic activity’ (23,001 genes; representing 49.8% of transcripts in the molecular function category), ‘binding’ (22,866 genes; 49.5%) and ‘transporter activity’ (2677 genes; 5.8%) were the most important sub-categories in the molecular function category (Additional file 4). In addition, a total of 23,211 transcripts were categorized into 175 pathways (Additional file 5), among which metabolic pathways, biosynthesis of secondary metabolites and ribosome pathways contained the most transcripts (Fig. 1b).

### Global Transcriptome changes

**Fig. 2:**
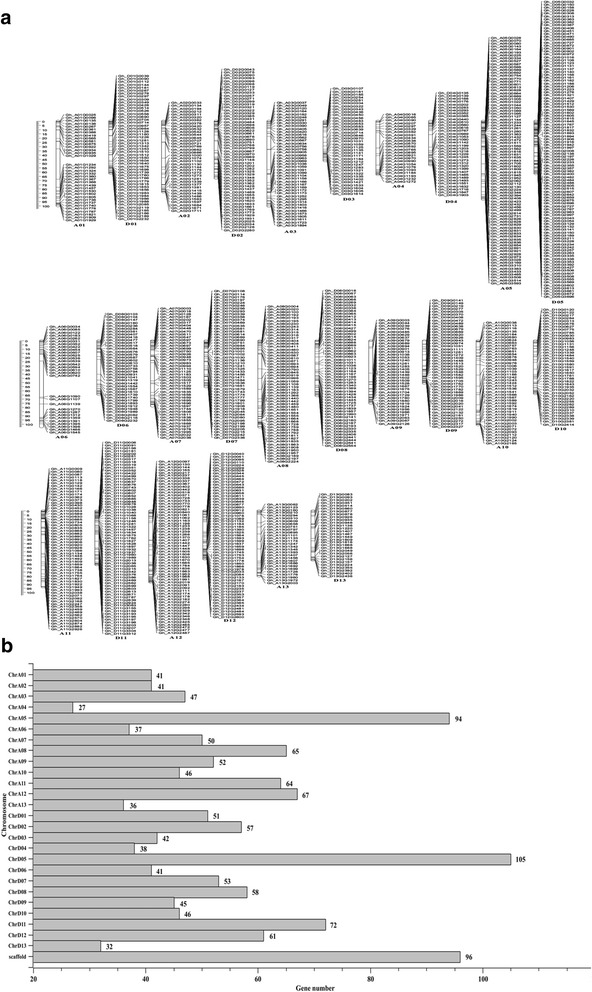
Distribution of the differentially expressed genes on different chromosomes. **a** Location distribution of DEGs on different chromosomes. **b** DEG numbers on different chromosomes. The Y-axis represents different chromosomes. xis and numbers behind each bar represent the DEG numbers on each chromosome

The number of reads mapped to the predicted transcripts of the TM-1 reference genome was calculated as the expression level for each gene. The following three comparisons of gene expression levels were performed: B (dB3) vs. A (dZB3), which had the isogenic nuclear genomes (containing the recessive non-functional *rf1* allele) but different cytoplasms and fertility; B (dB3) vs. R (dZK3), both of which were isogenic and fertile but differed in their cytoplasms and *Rf1* alleles; and A (dZB3) vs. R (dZK3), both of which had the same CMS-D2 cytoplasm but differed in fertility and *Rf1* alleles. A total of 728 (442 upregulated and 286 downregulated), 918 (524 upregulated and 394 downregulated) and 456 (176 upregulated and 280 downregulated) DEGs were identified in the above three comparisons, respectively (Additional files 6–8). These DEGs represented a total of 1464 non-redundant genes, including 1368 that were distributed across the 26 chromosomes of *G. hirsutum* and 96 genes on 56 scaffolds (Fig. 2). It is interesting to note that Chr_D05 (with restorer gene *Rf1*) and the homeologous Chr_A05 (99.5 DEGs vs. 48.7 DEGs) carried more DEGs than the other chromosomes. Furthermore, among the 1464 DEGs, three possible mitochondrial targeted protein-coding genes (*Gh_D01G1128*, *Gh_D06G0518* and *Gh_A03G1169*) and five possible chloroplast targeted protein- coding genes (*Gh_A13G2212*, *Gh_A05G2854*, *Gh_A12G0821*, *Gh_A12G0217* and *Gh_D11G3195*) were differentially expressed between dZK3 and dB3, and three possible chloroplast targeted protein-coding genes (*Gh_Sca078114G01*, *Gh_D01G0297* and Gh_A07G1517) were differentially expressed between dZB3 and dB3. These DEGs may be affected by the CMS-D2 cytoplasm.

**Fig. 3:**
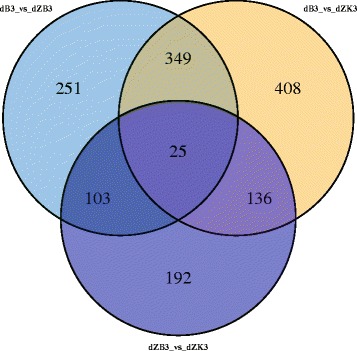
Venn diagram showing the distribution of unique and common DEGs among three comparisons

The distribution of unique and common DEGs for the three comparisons is shown in Fig. 3. The results indicated that 251 of 728 DEGs were unique to B (dB3) vs. A (dZB3), 408 of 918 were unique to B (dB3) vs. R (dZK3), and 192 of 456 were unique to A (dZB3) vs. R (dZK3). Compared with R (dZK3, containing the restorer gene), 136 common DEGs were identified in both B (dB3) and A (dZB3) containing the non-restoring gene. Compared with B (dB3, with normal Upland cotton cytoplasm), 349 common DEGs were identified in both A (dZB3) and R (dZK3), which contained the CMS-D2 cytoplasm. Compared with the male sterile A line (dZB3), 103 common DEGs were identified in the fertile B (dB3) and R (dZK3) lines.

### GO and KEGG enrichment analysis of DEGs

For the 728 DEGs between B (dB3) and A (dZB3), ‘metabolic process’, ‘catalytic activity’ and ‘single-organism process’ were the three most common GO terms (Additional file 9), and ‘metabolic pathways’, ‘biosynthesis of secondary metabolites’ and ‘microbial metabolism in diverse environments’ were the three most common KEGG pathways (Additional file 10). Seven DEGs associated with the GO terms ‘molecular transducer activity’ and ‘electron carrier activity’ were specifically upregulated and downregulated, respectively in dB3. For the 918 DEGs between B (dB3) and R (dZK3), ‘metabolic process’, ‘cellular process’ and ‘catalytic activity’ were the three most common GO terms (Additional file 11), while the three most common pathways (Additional file 12) were the same as in B (dB3) and A (dZB3). Six DEGs associated with the ‘cell junction’ and ‘symplast’ were specifically upregulated in R (dZK3). For the 456 DEGs between A (dZB3) and R (dZK3), ‘metabolic process’, ‘cellular process’ and ‘binding’ were the three most common GO terms (Additional file 13), and ‘metabolic pathways’, ‘biosynthesis of secondary metabolites’ and ‘drug metabolism cytochrome P450’ were the three most common pathways (Additional file 14). Eleven DEGs associated with growth, six with structural molecule activity and five with electron carrier activity were specific upregulated in dZB3.

To identify significant GO categories and KEGG pathways among the three comparisons, further GO and KEGG enrichment analyses were performed. The GO categories ‘negative regulation of circadian rhythm’, ‘transcription regulatory region DNA binding’ and ‘regulatory region nucleic acid binding’ had the highest enrichment ratios between the maintainer B line (dB3) and the CMS-D2 A (dZB3) line (Additional file 15), while ‘long-day photoperiodism’, ‘negative regulation of sequence-specific DNA binding transcription factor activity’ and ‘negative regulation of circadian rhythm’ had the highest enrichment ratios between the A line (dZB3) and the restorer R (dZK3) line (Additional file 16). ‘Allene-oxide cyclase activity’, ‘response to wounding’ and ‘oxidoreductase activity’ had the highest enrichment ratios between the B (dB3) and the R (dZK3) lines (Additional file 17).

The three primary KEGG pathways with the highest ratios were ‘circadian rhythm’, ‘alpha-linolenic acid metabolism’ and ‘sesquiterpenoid and triterpenoid biosynthesis’ between the B (dB3) and A (dZB3) lines (Additional file 18); ‘circadian rhythm’, ‘protein processing in endoplasmic reticulum’ and ‘photosynthesis’ between the A (dZB3) and R (dZK3) lines (Additional file 19); and ‘protein processing in endoplasmic reticulum’, ‘alpha-linolenic acid metabolism’ and ‘thyroid hormone synthesis’ between the B (dB3) and R (dZK3) lines (Additional file 20). The results showed that the circadian rhythm pathway was an important and common pathway that was affected during meiosis.

### Analysis of DEGs on Chr_D05 and DEGs related to circadian rhythms

**Fig. 4:**
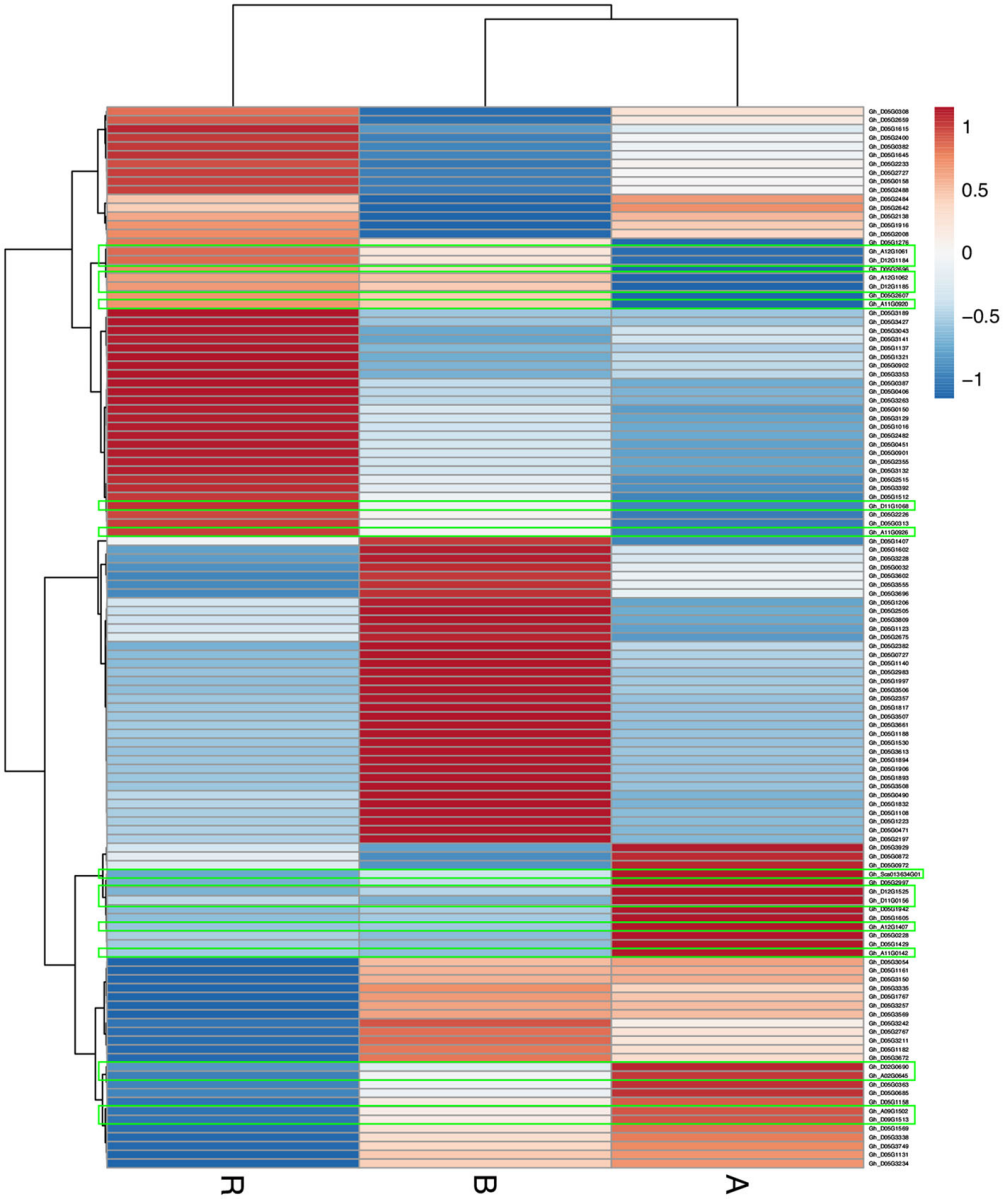
Heatmap showing the FPKM values of DEGs on Chr_D05 and DEGs related to circadian rhythm. The FPKM values for the DEGs in the three samples were used for hierarchical analysis. The heatmap shows the expression abundance of the DEGs. The colors correspond to FPKM values, ranging from blue (low expression) to red (high expression). Those genes in green boxes represent DEGs related to circadian rhythm

In our previous study [14], the restorer gene *Rf1* was shown to be located on Chr_D05 near position 54,287,522. In this study, *Gh_D05G3189* and *Gh_D05G3427* near the target region were found to be specifically expressed in the fertile R lines but were not expressed in the A or B lines. To further understand the effect of DEGs from regions adjacent to *Rf1*, GO enrichment analysis of 105 DEGs on Chr_D05 was performed. The results demonstrated that ‘sesquiterpene synthase activity’ and ‘(+)-delta-cadinene synthase activity’ were the two major GO terms with the highest enrichment ratios, while ‘sesquiterpenoid and triterpenoid biosynthesis’, ‘protein processing in endoplasmic reticulum’ and ‘carotenoid biosynthesis’ were the three major pathways identified in KEGG enrichment analysis. To examine the correlation between the expression of the DEGs in different samples, a heatmap analysis was performed based on the FPKM values of the 105 DEGs on Chr_D05 with the restorer gene and 16 DEGs related to the circadian rhythm (Fig. 4). The results showed that DEGs participating in sesquiterpene synthase activity and (+)-delta-cadinene synthase activity were all expressed preferentially in the B line, while most of the genes related to protein processing in the endoplasmic reticulum were highly expressed in the R line. Furthermore, it was interesting to find that most DEGs related to the circadian rhythm were highly expressed in the R and A lines with the CMS-D2 cytoplasm, implying a possible connection between the circadian rhythm and the CMS-D2 cytoplasm.

### Validation of RNA-seq data by qRT-PCR

**Fig. 5:**
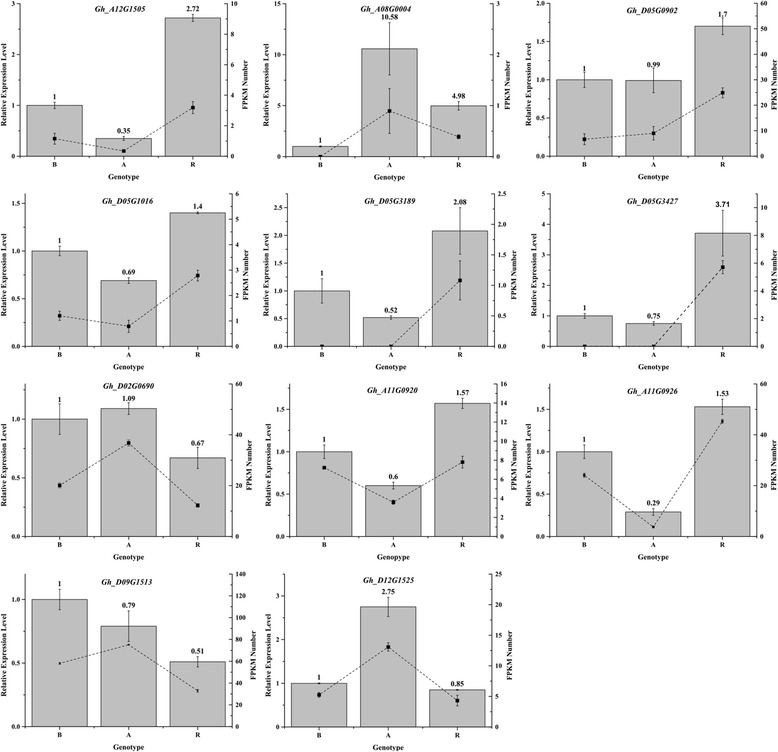
qRT-PCR analysis of gene expression compared with the RNA-seq data. The gray columns represented the relative expression levels of the genes; the dotted lines represent the RNA-seq reads. A: sterile line, B: maintainer line, R: restorer line

To validate the RNA-seq data using real-time qRT-PCR, 11 DEGs were selected based on high fold-changes (*Gh_A12G1505*), specific expression in certain genotypes (*Gh_A08G0004*), chromosomal location on Chr_D05 (*Gh_D05G0902, Gh_D05G1016, Gh_D05G3189,* and *Gh_D05G3427*), and association with the circadian rhythm (*Gh_D02G0690, Gh_A11G0920, Gh_A11G0926, Gh_D09G1513,* and *Gh_D12G1525*). The expression patterns of these genes are shown in Fig. 5. The results showed that except for the *Gh_D09G1513* gene, the expression patterns as determined by qRT-PCR were consistent with those obtained by RNA-seq, confirming the accuracy of the RNA-seq results in this study.

### SNP identification of DEGs on Chr_D05

The DEGs located on Chr_D05 with the restorer gene *Rf1* were chosen for identification of SNPs among the three lines (genotypes). For the 105 DEGs on Chr_D05, 11 SNP loci in 11 DEGs were identified between the sequences from the R line and those from the non-restoring genome, i.e., the A and B lines, including seven loci in exons and four loci downstream of the coding sequences (Table 1). Among these genes, *Gh_D05G3129*, *Gh_D05G3141*, *Gh_D05G3211* and *Gh_D05G3427* were located within the predicted target region of *Rf1*. Therefore, some of them may be related to the fertility-restoring gene, especially *Gh_D05G3427*, which is a proton pump-interactor 1-like gene that was expressed specifically in the restorer line.

**Table 1:** SNP information for DEGs on Chr_D05

*Gene*	Chromosome	Start	End	TM-1	Three line			Annotation	Mutationtype	Gene Annotation	fpkm		
					B	A	R				B	A	R
*Gh_D05G0901*	D05	7604314	7604314	A	A	A	C	downstream	•	17.3 kDa class I heat shock protein	17.4828	13.6636	29.6254
*Gh_D05G0972*	D05	8,177,118	8,177,118	A	A	A	G	exonic	•	probable aquaporin PIP2–2	1.85272	5.44079	3.00306
*Gh_D05G2138*	D05	20,020,319	20,020,319	T	T	T	C	downstream	•	protein DMR6-LIKE OXYGENASE 2-like	0.32569	1.99919	2.05069
*Gh_D05G2233*	D05	21,298,396	21,298,396	A	A	A	T	downstream	•	uncharacterized protein	7.05292	14.1268	20.0803
*Gh_D05G3043*	D05	40,631,403	40,631,403	G	G	G	A	exonic	•	lipid phosphate phosphatase 2-like	1.77645	2.15516	3.76462
*Gh_D05G3129*	D05	46,402,209	46,402,209	A	A	A	G	downstream	•	cytochrome P450 like_TBP	3.20327	1.11014	11.0941
*Gh_D05G3141*	D05	46,856,961	46,856,961	A	A	A	C	exonic	•	small ubiquitin-related modifier 1-like	14.5079	20.2293	40.4531
*Gh_D05G3211*	D05	49,817,512	49,817,512	T	T	T	A	exonic	•	elongation factor 2	59.3663	48.7858	26.7643
*Gh_D05G3427*	D05	55,765,423	55,765,423	A	A	A	T	exonic	•	proton pump-interactor 1-like	0	0	5.70722
*Gh_D05G3508*	D05	57,898,849	57,898,849	A	A	A	G	exonic	synonymous	(+)-delta-cadinene synthase	7.73005	0.79699	0.82779
*Gh_D05G3696*	D05	61,550,775	61,550,775	A	A	A	G	exonic	•	probable LRR receptor-like serine/threonine-protein kinase	1.43551	0.94060	0.59621

## Discussion

### Illumina sequencing and sequence annotation

The CMS system is considered the most important tool and is ideal for cotton hybrid seeds production. A restorer line containing a restorer gene is the determinant for the CMS system. Thus, to understand restorer genes, a large number of molecular mapping studies have been conducted. However, there have been no reports about how the restorer gene *Rf1* affects gene expression. In the present study, transcriptome sequencing was performed to generate large amounts of cDNA sequence data and profile transcriptome changes in a restorer gene backcross population (BC_8_F_1_) with CMS cytoplasm and its backcross parent (maintainer line) without the CMS-D2 cytoplasm. With the genome sequence of *G. hirsutum* used as the reference genome, more than 90% of clean reads were mapped to the reference genome. In total, 62,001 of the 70,478 predicted transcripts in the reference genome were identified in this study through gene annotation. Thus, the transcriptomic data in this study met the basic requirements needed for a comparative analysis. Finally, 1464 DEGs were identified among the three lines, many of which could serve as potential targets for future studies aimed at discovering the molecular mechanism of nucleo-cytoplasmic interactions.

### DEGs in the restorer Gene located on chromosome c5

The 1464 DEGs were mapped to 26 chromosomes and 56 scaffolds of *G. hirsutum*. Chr_D05 and its homeologous chromosome Chr_A05 were the two chromosomes with the most DEGs. In our previous study, the restorer gene *Rf1* was mapped to Chr_D05 [14]. This implied that the expression profiles of these genes may be affected by the restorer gene. Sesquiterpene synthase activity, (+)-delta-cadinene synthase activity and carotenoid biosynthesis were identified as important pathways according to the GO enrichment analysis of the 105 DEGs on Chr_D05. Cotton (+)-delta-cadinene synthase has been reported as a sesquiterpene cyclase that catalyzes a branch-point step leading to the biosynthesis of sesquiterpene phytoalexins, including gossypol [38–40]. In plants, carotenoids are crucial for various biological processes, such as photosynthesis, photoprotection, and regulation of growth and development [41–44], as well as responses to the environment [45, 46]. During field tests, the fertility of CMS-D2 restorer containing the restorer gene was affected by the environment. Therefore, whether there are correlations between terpene biosynthesis and functions of the restorer gene requires further study.

In our study, *Gh_D05G3427*, which had a SNP and specifically expression in the restorer line, was identified in the predicted target region of *Rf1* on Chr_D05. It is a proton pump-interactor 1-like gene (*PPI1*). Previous studies have demonstrated that the PPI1 is a novel protein that can interact with the C-terminal autoinhibitory domain of the plasma membrane (PM) H(+)-ATPase [47]. PM H + −ATPases are important for plant nutrient acquisition and can be detected at the whole plant level [48–50]. Furthermore, some PM H + −ATPases only expressed in anther tissues have been identified [51–53], implying that this type of genes is important for male gametogenesis. In this study, the PM H + −ATPases regulatory gene *Gh_D05G3427* was identified specifically in the restorer line. Thus, it could be a potentially important gene that interacts with the restorer gene and affects male gametophyte development. Further study of this gene is needed to elucidate the genetic and molecular mechanism of fertility restoration associated with *Rf1*.

### The circadian rhythm pathway and its relationship with pollen development

Previous research has shown that the circadian rhythm pathway is involved in the promotion of reproductive organs development in the vegetative stage in higher plants [54–56], photosynthesis [57, 58], starch metabolism [59–61], phytohormone response [61–63], hypocotyl elongation [64, 65], and plant–pathogen interaction [66]. Additionally, some research has indicated that the circadian rhythm pathway is involved in the male sterility transition [67, 68]. In this current study, several genes associated with the circadian rhythm were identified, some of which comprise interlocking transcriptional feedback loops that play important roles in the plant central clock. Some loops integrate environmental factors, such as light and temperature, into the central clock through the input signaling pathway and import the rhythm signal into downstream signaling pathways through output signaling pathways [69, 70]. Here, circadian rhythm differences between the fertile and sterile lines were also identified, and the differential expression profiles of the genes related to the circadian rhythm were confirmed by qRT-PCR. However, how the restorer gene regulates the circadian rhythm, which in turn regulates male fertility, needs a further study.

## Conclusions

Through genome-wide comparative transcriptome analysis, 1464 DEGs were identified in flower buds among the fertile R line, maintainer B line and sterile A line. The *Rf1*-carrying Chr_D05 and the homeologous Chr_A05 had more DEGs than the other chromosomes. qRT-PCR further confirmed the accuracy of the RNA-seq results. The circadian rhythm pathway was identified as an important pathway differing between the fertile and sterile lines by GO and KEGG enrichment analysis. In the predicted target region of *Rf1* on Chr_D05, *Gh_D05G3427* was found to be expressed specifically in the restorer line and to have a restorer line specific SNP. Our results provide useful data for future investigations into the molecular mechanisms of nucleo-cytoplasmic interaction in CMS cotton.

## Additional files

Additional file 1: Primers for quantitative RT-PCR (XLS 125 kb)

Additional file 2: Trimmed sequencing data (XLS 62 kb)

Additional file 3: Mapping percentage to the TM-1 reference genome (XLS 122 kb)

Additional file 4: GO classification of the expressed genes (XLS 60 kb)

Additional file 5: KEGG classification of the expressed genes (XLS 84 kb)

Additional file 6: Information on the differentially expressed genes between B and A (XLS 93 kb)

Additional file 7: Information on the differentially expressed genes between B and R (XLS 61 kb)

Additional file 8: Information on the differentially expressed genes between A and R (XLS 63 kb)

Additional file 9: GO analysis of DEGs between B and A (XLS 90 kb)

Additional file 10: KEGG analysis of DEGs between B and A (XLS 76 kb)

Additional file 11: GO analysis of DEGs between B and R (XLS 56 kb)

Additional file 12: KEGG analysis of DEGs between B and R (XLS 90 kb)

Additional file 13: GO analysis of DEGs between A and R (XLSX 41 kb)

Additional file 14: KEGG analysis of DEGs between A and R (XLS 56 kb)

Additional file 15: GO enrichment analysis of DEGs between B and A (XLS 68 kb)

Additional file 16: GO enrichment analysis of DEGs between A and R (XLS 2620 kb)

Additional file 17: GO enrichment analysis of DEGs between B and R (XLS 560 kb)

Additional file 18: KEGG enrichment analysis of DEGs between B and A (XLS 684 kb)

Additional file 19: KEGG enrichment analysis of DEGs between A and R (XLS 378 kb)

Additional file 20: KEGG enrichment analysis of DEGs between B and R (XLS 60 kb)

## Abbreviations

**AEP**: artificial emasculation and pollination
**CMS**: Cytoplasmic male sterility
**DEGs**: Differentially expressed genes
**FPKM**: Fragments per kilobase of exon per million fragments
**NGS**: Next-generation sequencing
**SNP**: Single nucleotide polymorphism

## References

[1] Yu S, Fan S, Wang H, Wei H, Pang C (2016). Progresses in research on cotton high yield breeding in China. Sci Agric Sin.

[2] Budar F, Pelletier G (2001). Male sterility in plants: occurrence, determinism, significance and use. C R Acad Sci III.

[3] Meyer VG (1975). Male sterility from *Gossypium harknessii*. J Hered.

[4] Weaver DB, Weaver JB (1977). Inheritance of pollen fertility restoration in cytoplasmic male-sterile upland cotton. Crop Sci.

[5] Zhang JF, Stewart JM (2001). CMS-D8 restoration in cotton is conditioned by one dominant gene. Crop Sci.

[6] Zhang JF, Stewart JM (2001). Inheritance and genetic relationships of the D8 and D2-2 restorer genes for cotton cytoplasmic male sterility. Crop Sci.

[7] Guo W, Zhang T, Pan J, Kohel R (1998). Identification of RAPD marker linked with fertility-restoring gene of cytoplasmic male sterile lines in upland cotton. Chin. Sci. Bull..

[8] Lan TH, Cook CG, Paterson AH. Identification of a RAPD marker linked to a male fertility restoration gene in cotton (*Gossypium hirsutum* L.). J Agric Genomics. 1999;4:1-5.

[9] Liu L, Go W, Zhu X (2003). Inheritance and fine mapping of fertility restoration for cytoplasmic male sterility in *Gossypium hirsutum* L. Theor. Appl. Genet..

[10] Feng CD, Stewart JM, Zhang JF (2005). STS markers linked to the *Rf1* fertility restorer gene of cotton. Theor. Appl. Genet..

[11] Yin J, Guo W, Yang L, Liu L, Zhang T (2006). Physical mapping of the *Rf1* fertility-restoring gene to a 100 kb region in cotton. Theor. Appl. Genet..

[12] Wang F, Yue B, Hu JG, Stewart JM, Zhang JF (2009). A target region amplified polymorphism marker for fertility restorer gene *Rf(1)* and chromosomal localization of *Rf(1)* and *Rf(2)* in cotton. Crop Sci.

[13] Yang L. Map-based cloning of fertility restoring gene of CMS and analysis of PPR gene family in cotton. Nanjin: Nanjin Agricultural University; 2009.

[14] Wu J, Cao X, Guo L, Qi T, Wang H, Tang H, et al. Development of a candidate gene marker for *Rf1* based on a PPR gene in cytoplasmic male sterile CMS-D2 upland cotton. Mol Breed. 2014:1–10.

[15] Mutz K-O, Heilkenbrinker A, Lönne M, Walter J-G, Stahl F (2013). Transcriptome analysis using next-generation sequencing. Curr. Opin. Biotechnol..

[16] Pang M, Percy RG, Stewart JM, Hughs E, Zhang J (2012). Comparative transcriptome analysis of pima and Acala cotton during boll development using 454 pyrosequencing technology. Mol Breed.

[17] Yoo M-J, Wendel JF (2014). Comparative evolutionary and developmental dynamics of the cotton (*Gossypium hirsutum*) fiber transcriptome. PLoS Genet..

[18] Naoumkina M, Thyssen GN, Fang DD (2015). RNA-seq analysis of short fiber mutants Ligon-lintless-1 (Li1) and −2 (Li2) revealed important role of aquaporins in cotton (*Gossypium hirsutum* L.) fiber elongation. BMC Plant Biol.

[19] Islam MS, Fang DD, Thyssen GN, Delhom CD, Liu Y, Kim HJ (2016). Comparative fiber property and transcriptome analyses reveal key genes potentially related to high fiber strength in cotton (*Gossypium hirsutum* L.) line MD52ne. BMC Plant Biol.

[20] Lin M, Pang C, Fan S, Song M, Wei H, Yu S (2015). Global analysis of the *Gossypium hirsutum* L. transcriptome during leaf senescence by RNA-Seq. BMC Plant Biol.

[21] Tao T, Zhao L, Lv Y, Chen J, Hu Y, Zhang T (2013). Transcriptome sequencing and differential gene expression analysis of delayed gland morphogenesis in *Gossypium australe* during seed germination. PLoS One.

[22] Bowman MJ, Park W, Bauer PJ, Udall JA, Page JT, Raney J (2013). RNA-Seq transcriptome profiling of upland cotton (*Gossypium hirsutum* L.) root tissue under water-deficit stress. PLoS One.

[23] Zhang X, Yao D, Wang Q, Xu W, Wei Q, Wang C (2013). mRNA-seq analysis of the *Gossypium arboreum* transcriptome reveals tissue selective signaling in response to water stress during seedling stage. PLoS One.

[24] Zhang F, Zhu G, Du L, Shang X, Cheng C, Yang B (2016). Genetic regulation of salt stress tolerance revealed by RNA-Seq in cotton diploid wild species, *Gossypium davidsonii*. Sci. Rep..

[25] Xu L, Zhu L, Tu L, Liu L, Yuan D, Jin L (2011). Lignin metabolism has a central role in the resistance of cotton to the wilt fungus *Verticillium dahliae* as revealed by RNA-Seq-dependent transcriptional analysis and histochemistry. J. Exp. Bot..

[26] Artico S, Ribeiro-Alves M, Oliveira-Neto OB, de Macedo LLP, Silveira S, Grossi-de-Sa MF (2014). Transcriptome analysis of *Gossypium hirsutum* flower buds infested by cotton boll weevil (*Anthonomus grandis*) larvae. BMC Genomics.

[27] Suzuki H, Yu J, Ness SA, O’Connell MA, Zhang J (2013). RNA editing events in mitochondrial genes by ultra-deep sequencing methods: a comparison of cytoplasmic male sterile, fertile and restored genotypes in cotton. Mol Genet Genomics MGG.

[28] Fang W, Zhao F, Sun Y, Xie D, Sun L, Xu Z (2015). Transcriptomic profiling reveals complex molecular regulation in cotton genic male sterile mutant Yu98-8A. PLoS One.

[29] Zhang J, Turley RB, Stewart JM (2008). Comparative analysis of gene expression between CMS-D8 restored plants and normal non-restoring fertile plants in cotton by differential display. Plant Cell Rep..

[30] Suzuki H, Rodriguez-Uribe L, Xu J, Zhang J (2013). Transcriptome analysis of cytoplasmic male sterility and restoration in CMS-D8 cotton. Plant Cell Rep..

[31] Wang K, Wang Z, Li F, Ye W, Wang J, Song G (2012). The draft genome of a diploid cotton *Gossypium raimondii*. Nat. Genet..

[32] Paterson AH, Wendel JF, Gundlach H, Guo H, Jenkins J, Jin D (2012). Repeated polyploidization of *Gossypium* genomes and the evolution of spinnable cotton fibres. Nature.

[33] Li F, Fan G, Wang K, Sun F, Yuan Y, Song G (2014). Genome sequence of the cultivated cotton *Gossypium arboreum*. Nat. Genet..

[34] Li F, Fan G, Lu C, Xiao G, Zou C, Kohel RJ (2015). Genome sequence of cultivated upland cotton (*Gossypium hirsutum* TM-1) provides insights into genome evolution. Nat. Biotechnol..

[35] Zhang T, Hu Y, Jiang W, Fang L, Guan X, Chen J (2015). Sequencing of allotetraploid cotton (*Gossypium hirsutum* L. acc. TM-1) provides a resource for fiber improvement. Nat. Biotechnol..

[36] Trapnell C, Pachter L, Salzberg SL (2009). TopHat: discovering splice junctions with RNA-Seq. Bioinformatics.

[37] Xie C, Mao X, Huang J, Ding Y, Wu J, Dong S (2011). KOBAS 2.0: a web server for annotation and identification of enriched pathways and diseases. Nucleic Acids Res..

[38] Tan XP, Liang WQ, Liu CJ, Luo P, Heinstein P, Chen XY (2000). Expression pattern of (+)-delta-cadinene synthase genes and biosynthesis of sesquiterpene aldehydes in plants of *Gossypium arboreum* L. Planta.

[39] Xu Y-H, Wang J-W, Wang S, Wang J-Y, Chen X-Y (2004). Characterization of *GaWRKY1*, a cotton transcription factor that regulates the sesquiterpene synthase gene (+)-delta-cadinene synthase-a. Plant Physiol..

[40] Ma D, Hu Y, Yang C, Liu B, Fang L, Wan Q (2016). Genetic basis for glandular trichome formation in cotton. Nat. Commun..

[41] Cazzonelli CI, Pogson BJ (2010). Source to sink: regulation of carotenoid biosynthesis in plants. Trends Plant Sci..

[42] Ruiz-Sola MÁ, Rodríguez-Concepción M (2012). Carotenoid biosynthesis in *Arabidopsis*: a colorful pathway. Arab Book.

[43] Havaux M (2014). Carotenoid oxidation products as stress signals in plants. Plant J Cell Mol Biol.

[44] Nisar N, Li L, Lu S, Khin NC, Pogson BJ (2015). Carotenoid metabolism in plants. Mol. Plant.

[45] Walter MH, Strack D (2011). Carotenoids and their cleavage products: biosynthesis and functions. Nat. Prod. Rep..

[46] Cazzonelli CI (2011). Goldacre review: Carotenoids in nature: insights from plants and beyond. Funct Plant Biol.

[47] Bonza MC, Fusca T, Homann U, Thiel G, De Michelis MI (2009). Intracellular localisation of PPI1 (proton pump interactor, isoform 1), a regulatory protein of the plasma membrane H(+)-ATPase of *Arabidopsis thaliana*. Plant Biol Stuttg Ger.

[48] Arango M, Gévaudant F, Oufattole M, Boutry M (2003). The plasma membrane proton pump ATPase: the significance of gene subfamilies. Planta.

[49] Palmgren MG (2001). Plant plasma membrane H+−ATPases: powerhouses for nutrient uptake. Annu Rev Plant Physiol Plant Mol Biol.

[50] Sondergaard TE, Schulz A, Palmgren MG (2004). Energization of transport processes in plants. Roles of the plasma membrane H+−ATPase. Plant Physiol..

[51] Lefebvre B, Arango M, Oufattole M, Crouzet J, Purnelle B, Boutry M (2005). Identification of a *Nicotiana plumbaginifolia* plasma membrane H(+)-ATPase gene expressed in the pollen tube. Plant Mol. Biol..

[52] Houlné G, Boutry M (1994). Identification of an *Arabidopsis thaliana* gene encoding a plasma membrane H(+)-ATPase whose expression is restricted to anther tissue. Plant J Cell Mol Biol.

[53] Bock KW, Honys D, Ward JM, Padmanaban S, Nawrocki EP, Hirschi KD (2006). Integrating membrane transport with male gametophyte development and function through transcriptomics. Plant Physiol..

[54] McClung CR (2006). Plant circadian rhythms. Plant Cell.

[55] Turck F, Fornara F, Coupland G (2008). Regulation and identity of florigen: flowering locus T moves center stage. Annu. Rev. Plant Biol..

[56] Kobayashi Y, Weigel D (2007). Move on up, it’s time for change--mobile signals controlling photoperiod-dependent flowering. Genes Dev..

[57] Dodd AN, Salathia N, Hall A, Kévei E, Tóth R, Nagy F (2005). Plant circadian clocks increase photosynthesis, growth, survival, and competitive advantage. Science.

[58] Yakir E, Hilman D, Harir Y, Green RM (2007). Regulation of output from the plant circadian clock. FEBS J.

[59] McClung CR, Gutiérrez RA (2010). Network news: prime time for systems biology of the plant circadian clock. Curr. Opin. Genet. Dev..

[60] de Montaigu A, Tóth R, Coupland G (2010). Plant development goes like clockwork. Trends Genet TIG.

[61] Doherty CJ, Kay SA (2010). Circadian control of global gene expression patterns. Annu. Rev. Genet..

[62] Covington MF, Maloof JN, Straume M, Kay SA, Harmer SL (2008). Global transcriptome analysis reveals circadian regulation of key pathways in plant growth and development. Genome Biol.

[63] Michael TP, Breton G, Hazen SP, Priest H, Mockler TC, Kay SA (2008). A morning-specific phytohormone gene expression program underlying rhythmic plant growth. PLoS Biol..

[64] Nozue K, Covington MF, Duek PD, Lorrain S, Fankhauser C, Harmer SL (2007). Rhythmic growth explained by coincidence between internal and external cues. Nature.

[65] Niwa Y, Yamashino T, Mizuno T (2009). The circadian clock regulates the photoperiodic response of hypocotyl elongation through a coincidence mechanism in *Arabidopsis thaliana*. Plant Cell Physiol..

[66] Roden LC, Ingle RA (2009). Lights, rhythms, infection: the role of light and the circadian clock in determining the outcome of plant-pathogen interactions. Plant Cell.

[67] Wang W, Liu Z, Guo Z, Song G, Cheng Q, Jiang D (2011). Comparative transcriptomes profiling of photoperiod-sensitive male sterile rice Nongken 58S during the male sterility transition between short-day and long-day. BMC Genomics.

[68] Hu J, Chen X, Zhang H, Ding Y (2015). Genome-wide analysis of DNA methylation in photoperiod- and thermo-sensitive male sterile rice Peiai 64S. BMC Genomics.

[69] Locke JCW, Southern MM, Kozma-Bognár L, Hibberd V, Brown PE, Turner MS (2005). Extension of a genetic network model by iterative experimentation and mathematical analysis. Mol Syst Biol.

[70] Locke JCW, Kozma-Bognár L, Gould PD, Fehér B, Kevei E, Nagy F (2006). Experimental validation of a predicted feedback loop in the multi-oscillator clock of *Arabidopsis thaliana*. Mol Syst Biol.

